# Characterization of the complete chloroplast genome of *Xanthium sibiricum*, one of the traditional Chinese medicines in China

**DOI:** 10.1080/23802359.2020.1731368

**Published:** 2020-02-28

**Authors:** Jing Lv, Shibing Zhu, Shan Liu

**Affiliations:** a Department of Internal Medicine, Zhejiang Chinese Medicine and Western Medicine Integrated Hospital, Hangzhou, Zhejiang, China;; b Basic Medical College, Zhejiang Chinese Medical University, Hangzhou, Zhejiang, China

**Keywords:** *Xanthium sibiricum*, Asteraceae, chloroplast genome, phylogenetic relationships, evolutionary relationship

## Abstract

*Xanthium sibiricum* is a common and annual the Traditional Chinese Medicine for many years in China. In this study, we presented the complete chloroplast genome of *X. sibiricum*. The chloroplast genome size was 151,897 bp in length that contained a large single-copy region (LSC) of 83,847 bp, a small single-copy region (SSC) of 17,890 bp and two inverted repeat regions (IR) of 25,080 bp. The overall nucleotide composition of the chloroplast genome is: 31.2% of A, 31.3% T, 18.5% C and 19.0% G, with a total G + C content of the chloroplast genome 37.5% and A + T of 62.5%. The chloroplast genome of *X. sibiricum* contains 133 genes, which included 88 protein-coding genes (PCG), 37 transfer RNA (tRNAs), and 8 ribosome RNA (rRNAs). Phylogenetic neighbour-joining (NJ) analysis result shown that the position of *X. sibiricum* closely related to *X. strumarium* in evolutionary relationship.

*Xanthium sibiricum* belongs to the family Asteraceae, and named Cang-Er in Chinese language. *Xanthium sibiricum* is used to dispel pathogenic wind and cold, relieve stuffy nose, expel wind and damp and relieve itching skin. It is also used to cure nasal running, headache caused by wind-cold, syndrome due to wind-damp, nettle rash, eczema and acariasis (Shi et al. 2015). Plant chloroplast genome is used for plant species identification in evolutionary studies, providing large amounts of information regarding genetics, taxonomy and phylogeny because of their relatively conserved gene structure and sequence divergence between species (Lima et al. 2016). In this study, we presented the complete chloroplast genome of *Xanthium sibiricum*, which can be useful for studying the medicinal valuable and the drug development, which also will provide more information that can assist in genome-wide evolutionary research in future.

The fresh specimen sample of *X. sibiricum* was collected from herb market near Zhejiang Chinese Medical University that located at Hangzhou, Zhejiang, China (119.89E, 30.09 N). The total genomic DNA of *X. sibiricum* was extracted using Plant Tissues Genomic DNA Extraction Kit (TaKaRa, DL, and CN) and deposited at Zhejiang Chinese Medical University (No. ZJCMU-001). The genomic DNA of *X. sibiricum* was purified and fragmented using the NEB Next Ultra^TM^ II DNA Library Prep Kit (NEB, BJ, CN) that the chloroplast genome was sequenced. Adapters control was performed and removed low-quality reads using the NGS QC Toolkit software (Patel and Jain 2012). The chloroplast genome was assembled and annotated using the MitoZ software (Meng et al. 2019). The physical map of the assembled chloroplast genome was generated using OGDRAW (Lohse et al. 2013).

The complete chloroplast genome of *X. sibiricum* (MK9845982) was 151,897 bp in size that contained a large single-copy region (LSC) of 83,847 bp, a small single-copy region (SSC) of 17,890 bp, and two inverted repeat regions (IR) of 25,080 bp. The cp genome of *X. sibiricum* comprised 133 genes that included 88 protein-coding genes (PCG), 37 transfer RNA genes (tRNA), and 8 ribosomal RNA genes (rRNA). In one of the IR, 19 genes were found duplicated, including 8 PCG species, 7 tRNA species, and 4 rRNA species. The overall nucleotide composition of the chloroplast genome is: 31.2% of A, 31.3% T, 18.5% C, and 19.0% G that is the total G + C content of 37.5% and A + T of 62.5%.

For phylogenetic analysis using the neighbour-joining (NJ) methods, we analyzed the relationship of 12 plant species chloroplast genomes with *X. sibiricum*. The phylogenetic tree was reconstructed using NJ methods and was performed using RAxML software (Stamatakis 2014) with the most suitable model. Branch support was inferred using 2000 bootstrap replicates. The phylogenetic tree was represented using MEGA X (Kumar et al. 2018) and edited using Figtree version 1.4.4. Furthermore, the phylogenetic NJ tree result showed that the chloroplast genome of *X. sibiricum* is clustered and closest to *X. strumarium* in the evolutionary relationship (Figure 1). This study can be useful for study the medicinal valuable and the drug development for this species in future.

**Figure 1.**
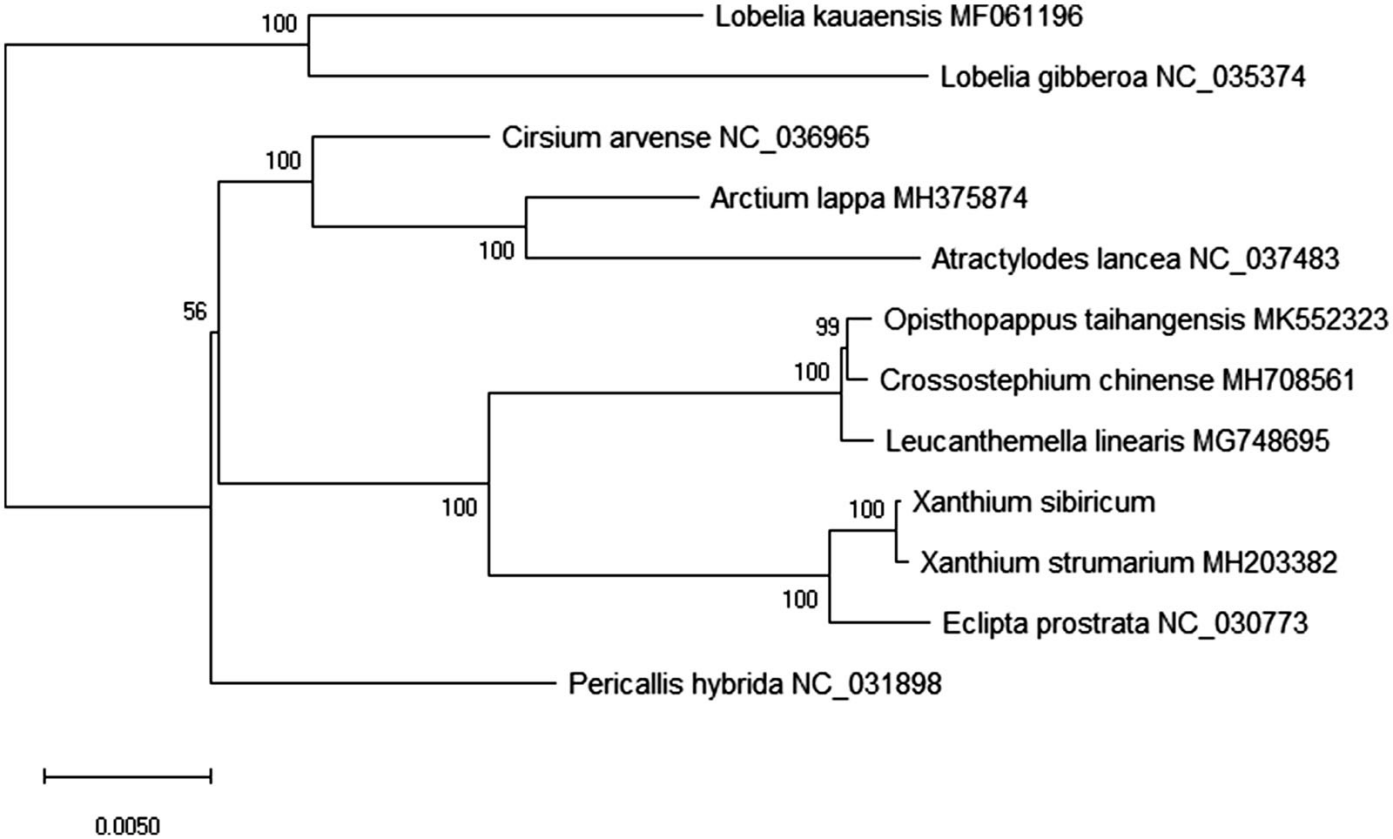
The phylogenetic NJ tree based on the 12 plant species complete chloroplast genome sequence that bootstrap repeat was 2000. The length of branch represents the divergence distance. All the plant species chloroplast genomes in this study have been deposited in the GenBank.
